# HPLC-UV, MALDI-TOF-MS and ESI-MS/MS Analysis of the Mechlorethamine DNA Crosslink at a Cytosine-Cytosine Mismatch Pair

**DOI:** 10.1371/journal.pone.0020745

**Published:** 2011-06-06

**Authors:** Pornchai Rojsitthisak, Nutthapon Jongaroonngamsang, Rebecca M. Romero, Ian S. Haworth

**Affiliations:** 1 Department of Food and Pharmaceutical Chemistry, Faculty of Pharmaceutical Sciences, Chulalongkorn University, Bangkok, Thailand; 2 Department of Pharmacology and Pharmaceutical Sciences, University of Southern California, Los Angeles, California, United States of America; St. Georges University of London, United Kingdom

## Abstract

**Background:**

Mechlorethamine [ClCH_2_CH_2_N(CH_3_)CH_2_CH_2_Cl], a nitrogen mustard alkylating agent, has been proven to form a DNA interstrand crosslink at a cytosine-cytosine (C-C) mismatch pair using gel electrophoresis. However, the atomic connectivity of this unusual crosslink is unknown.

**Methodology/Principal Findings:**

HPLC-UV, MALDI-TOF-MS, and ESI-MS/MS were used to determine the atomic connectivity of the DNA C-C crosslink formed by mechlorethamine, MALDI-TOF-MS of the HPLC-purified reaction product of mechlorethamine with the DNA duplex d[CTCACACCGTGGTTC]•d[GAACCACCGTGTGAG] (underlined bases are a C-C mismatch pair) indicated formation of an interstrand crosslink at m/z 9222.088 [M−2H+Na]^+^. Following enzymatic digestion of the crosslinked duplex by snake venom phosphodiesterase and calf intestinal phosphatase, ESI-MS/MS indicated the presence of dC-mech-dC [mech = CH_2_CH_2_N(CH_3_)CH_2_CH_2_] at m/z 269.2 [M]^2+^ (expected m/z 269.6, exact mass 539.27) and its hydrolytic product dC-mech-OH at m/z 329.6 [M]^+^ (expected m/z 329.2). Fragmentation of dC-mech-dC gave product ions at m/z 294.3 and 236.9 [M]^+^, which are both due to loss of the 4-amino group of cytosine (as ammonia), in addition to dC and dC+HN(CH_3_)CH = CH_2_, respectively. The presence of m/z 269.2 [M]^2+^ and loss of ammonia exclude crosslink formation at cytosine N^4^ or O^2^ and indicate crosslinking through cytosine N^3^ with formation of two quaternary ammonium ions.

**Conclusions:**

Our results provide an important addition to the literature, as the first example of the use of HPLC and MS for analysis of a DNA adduct at the N^3^ position of cytosine.

## Introduction

DNA damage and mutation can have major effects on genetic information that may alter the function of essential proteins and cause disease. Mismatching of paired bases is a common type of DNA damage that can result in harmful mutations and a specific DNA mismatch repair mechanism is required for this damage [1]. Sources of mismatch base pairs include replication errors due to direct misincorporation of bases, lesions in the parent strand, and formation of a heteroduplex between two homologous DNA molecules during recombination [2]–[4]. Mismatches may also be generated in hairpins formed by trinucleotide repeat sequences [5]–[7]. Mismatch base pairs cause thermodynamic instability of DNA duplexes [8], but most retain an intrahelical conformation with one or more hydrogen bonds between the bases: examples include A-A [9], G-G [9], A-C [10], G-T [11], C-T [12] and C-C [12]–[14] pairs. Establishment of DNA structures containing mismatch base pairs is important for understanding their involvement in replication, repair, and recombination.

The antiparallel C-C pair is one of the least stable mismatch pairs [8]. This instability causes the C-C pair to adopt different conformations, including both intra- and extrahelical positions [12]–[18]. Gao et al. first showed that a C-C mismatch pair could adopt an extrahelical conformation in a DNA duplex [15], whereas Boulard et al. described an intrahelical C-C mismatch pair with a single hydrogen bond, but also noted the flexibility and apparent pH dependence of the conformation [12]. In the d[CCG]_n_ triplet repeat hairpin, a C-C mismatch pair is present as every third base pair of the stem [16]–[18]. In such hairpins and equivalent duplexes, the C-C mismatch seems to be mainly intrahelical and hydrogen bonded [13], [14]. However, the d[CCG]_15_ hairpin shows pH-dependent electrophoretic mobility, which led to the proposal that multiple C-C mismatch pairs in this hairpin might partly adopt extrahelical locations [18]. The structure of this hairpin may also be of interest from a disease perspective, since the d[CCG]_n_ triplet repeat sequence is present in the d[CGG]_n_·d[CCG]_n_ genomic region that is expanded in Fragile X syndrome [6], [7]. It has been suggested that both strands of this sequence form hairpins, and that these hairpins are important in the mechanism of repeat expansion and consequently in development of the disease [13]–[21].

The dynamic properties of d[CCG]_n_ hairpins and their potential importance in Fragile X syndrome led us to seek a chemical agent that could probe these structures. In the course of this work, we made the unexpected discovery that mechlorethamine [ClCH_2_CH_2_N(CH_3_)CH_2_CH_2_Cl], a nitrogen mustard alkylating agent, is able to form a DNA interstrand crosslink at a C-C mismatch pair [19]–[21], with this crosslink forming in preference to the better known 1,3 G-G interstrand crosslink of mechlorethamine [22], [23]. We proposed that the mechlorethamine C-C crosslink forms in the DNA minor groove through the N^3^ of cytosine [19], but at the time we were unable to prove the atomic connectivity of the crosslink.

High performance liquid chromatography (HPLC) has been used widely for detection and purification of DNA adducts and crosslinks [22]–[26], and characterization of DNA adducts and crosslinks has been achieved using HPLC coupled with mass spectrometry (MS) [27]–[43]. Several of these studies have used MALDI-TOF-MS for determination of the molecular weight of the crosslinked duplex, followed by enzymatic digestion to obtain a fragment containing the specific crosslinked nucleosides for comparison with a synthetic standard [31]–[33]. Noll et al. used this approach for a duplex containing a synthetic N^4^dC-ethyl-N^4^dC crosslink [31] and these techniques have similarly been applied for N^3^dT-alkyl-N^3^dT and O^6^dG-heptyl-O^6^dG crosslinks [32], [33].

In ESI-MS/MS analysis using the product ion scan mode, a molecular ion detected from the initial electrospray ionization is fragmented by collision-induced dissociation (CID)-MS [28]–[30]. In this mode, molecular ions with m/z corresponding to the expected molecular weight of a crosslinked DNA species can be selected for fragmentation. The resulting product ions are then used to interpret the structure of the molecular ion [34], [35]. ESI-MS/MS has been used to determine the atomic connectivity of crosslinks formed by mechlorethamine at A and G [34] and to study G-G crosslinking by 1,2,3,4-diepoxybutane [36], [37] and 1,3-butadiene [38]. In this study, we used HPLC purification, MALDI-TOF-MS, enzymatic digestion and ESI-MS/MS to determine the connectivity of the DNA C-C crosslink formed by mechlorethamine.

## Results

### Characterization of the mechlorethamine-crosslinked DNA duplex by HPLC and MALDI-TOF-MS

**Figure 1:**
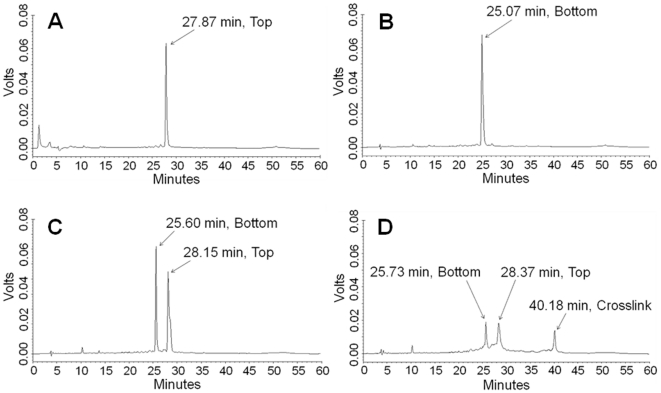
HPLC-UV chromatograms of DNA and the mechlorethamine-crosslinked DNA duplex. **A:** Top-strand DNA. **B:** Bottom-strand DNA. **C:** DNA duplex (denatured on the column). **D:** The mechlorethamine-crosslinked DNA duplex.

HPLC chromatograms of top- and bottom-strand DNA gave single peaks at 27.87 and 25.07 min, respectively (Figures 1A & 1B). The chromatogram of the annealed duplex gave two peaks at 25.60 and 28.15 min (Figure 1C) due to denaturation of the duplex on the column. The chromatogram of the crosslinked DNA (Figure 1D) showed three peaks at retention times of 25.73, 28.37 and 40.18 min. The first two of these peaks correspond to bottom-strand and top-strand DNA, respectively. The third peak was tentatively assigned to the mechlorethamine-DNA crosslinked duplex and comprised 25% of the total peak area.

**Figure 2:**
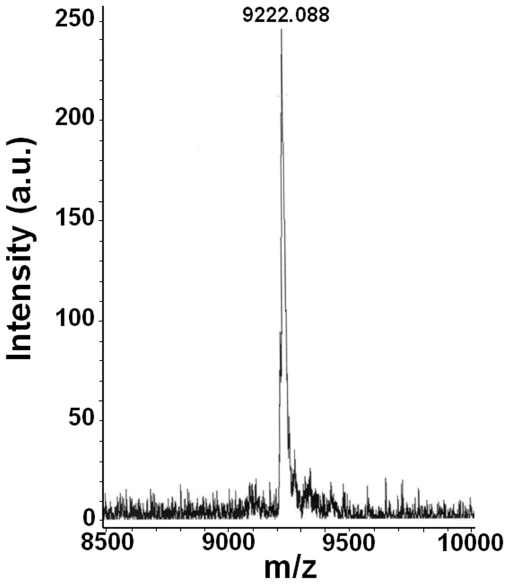
MALDI-TOF-MS spectrum of the mechlorethamine-crosslinked DNA duplex.

Following purification and desalting of the eluent collected at 40.18 min, the eluted sample was analyzed by MALDI-TOF-MS and a signal with [M−2H+Na]^+^ m/z 9222.088 (expected [M−2H+Na]^+^ m/z 9214.2) was detected (Figure 2). The difference between the observed and expected m/z was about 7.9 amu, giving a mass difference of 0.08% or a mass accuracy of 99.92%. Mass-accuracy measurements of large oligonucleotides can have errors of 0.3% [44]–[45] due to the instability of mixed-base oligodeoxynucleotides and metal ion contamination [30], [46]–[48]. DNA analysis by MALDI-TOF-MS in the presence of metal contaminants such as sodium and potassium may show degraded performance including peak broadening and reduced mass resolution, sensitivity and accuracy due to interacion between cations and the negatively charged sugar-phosphate backbone [30]. Therefore, the broad signal and the slight discrepancy between the observed and expected m/z may be due to interaction of cations with the DNA. The cations could be contaminants from salts and buffers used in the preparation and purification of the crosslinked duplexes.

### Enzymatic digestion of the mechlorethamine-crosslinked DNA duplex

**Figure 3:**
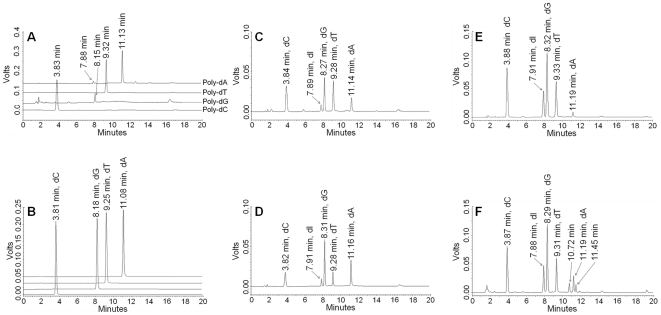
HPLC-UV chromatograms of monodeoxynucleosides. **A:** SVPD and CIP-digested poly-dC, poly-dG, poly-dT and poly-dA. **B:** Standard dC, dG, dT and dA. **C:** SVPD- and CIP-digested products of top-strand DNA. **D:** SVPD- and CIP-digested products of bottom-strand DNA. **E:** SVPD- and CIP-digested products of DNA duplex. **F:** SVPD- and CIP-digested products of the mechlorethamine-crosslinked DNA duplex.

HPLC chromatograms of the SVPD and CIP-digested products of poly-dC, poly-dG, poly-dT and poly-dA showed peaks at 3.83, 8.15, 9.32 and 11.13 min (Figure 3A). These corresponded to the standard monodeoxynucleosides dC, dG, dT and dA, which had retention times of 3.81, 8.18, 9.25 and 11.08 min, respectively (Figure 3B). Enzymatic digestion of top-strand DNA, bottom-strand DNA and the DNA duplex gave similar chromatograms with four peaks at retention times of about 3.8, 8.3, 9.3 and 11.2 min (Figure 3C–E). These chromatograms also showed an additional peak at a retention time of 7.9 min, which is a deoxyinosine peak resulting from deamination of dA due to the contamination of adenine deaminase in the snake venom phosphodiesterase, as previously reported by Wilds et al [33]. This conclusion was also supported by the observation that this peak appeared only on the chromatogram of the SVPD and CIP-digested products of poly-dA, but not for poly-dC, poly-dG and poly-dT (Figure 3A). The deoxyinosine peak and the four mononucleoside peaks were also observed after digestion of the mechlorethamine-crosslinked duplex, together with two additional peaks at retention times of 10.72 and 11.45 min (Figure 3F).

### ESI-MS/MS analysis of the atomic connectivity of the mechlorethamine C-C crosslink

**Figure 4:**
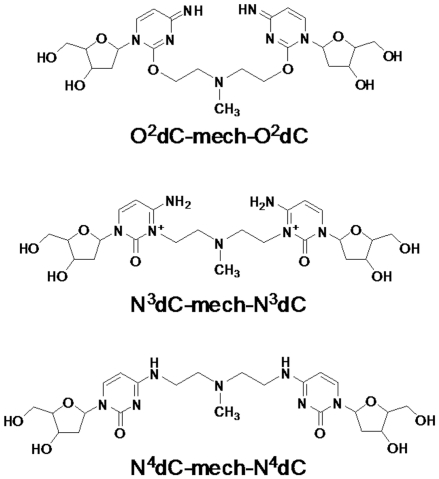
Structures of possible mechlorethamine crosslinks at a cytosine-cytosine mismatch pair. **A:** A mechlorethamine crosslink through O^2^ of cytosine. **B:** A mechlorethamine crosslink through N^3^ of cytosine. **C:** A mechlorethamine crosslink through N^4^ of cytosine. Note that the O^2^ and N^4^ crosslinks are neutral species that form with loss of 2H^+^ from the nucleosides, whereas the N^3^ crosslink has a double positive charge.

The products of digestion of the mechlorethamine-crosslinked duplex were analyzed by ESI-MS/MS. Molecular ions with m/z corresponding to possible crosslink species were selected for fragmentation. In this process, mechlorethamine crosslinks forming through the O^2^, N^3^ and N^4^ atoms of cytosine were considered (Figure 4). The O^2^dC-mech-O^2^dC and N^4^dC-mech-N^4^dC crosslinks [mech = CH_2_CH_2_N(CH_3_)CH_2_CH_2_] are neutral and should give a [M+H]^+^ molecular ion of m/z 538.2. In contrast, the N^3^dC-mech-N^3^dC crosslink has a double positive charge (Figure 4) and should give a [M]^2+^ molecular ion with m/z 269.6.

**Figure 5:**
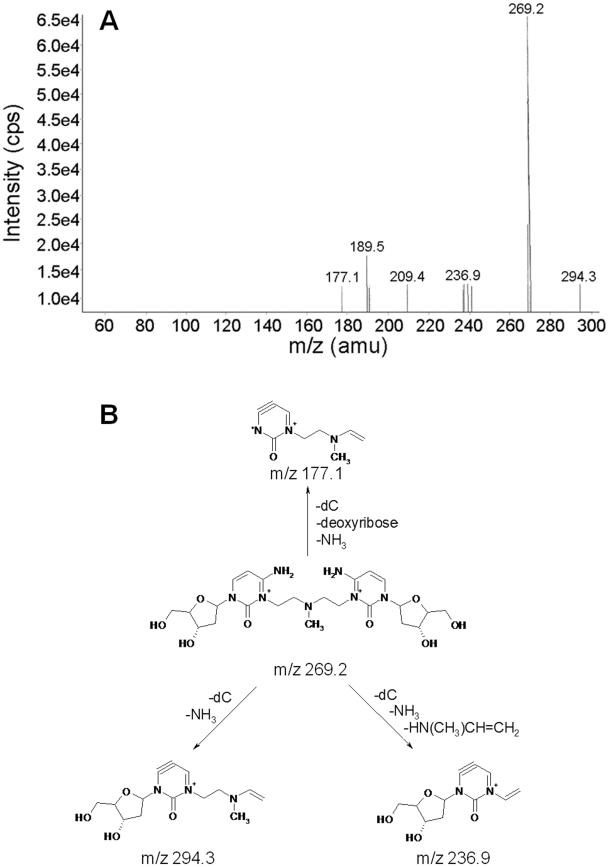
ESI-MS/MS analysis of N^3^dC-mech-N^3^dC. **A:** ESI-MS/MS spectrum of N^3^dC-mech-N^3^dC. **B:** Fragmentation pattern of N^3^dC-mech-N^3^dC. The IUPAC name for N^3^dC-mech-N^3^dC is [4-amino-3-[2-[2-[6-amino-3-[4-hydroxy-5-(hydroxymethyl)tetrahydrofuran-2-yl]-2-oxo-pyrimidin-1-ium-1-yl]ethyl-methyl-amino]ethyl]-1-[4-hydroxy-5-(hydroxymethyl)tetrahydrofuran-2-yl]pyrimidin-3-ium-2-one].

No signals were present in a range of m/z 538±5, but a signal with m/z 269.2 was detected. Fragmentation of [M]^2+^ m/z 269.2 gave product ions with m/z 177.1, 189.5, 209.4, 236.9 and 294.3 (Figure 5). The ion at m/z 294.3 occurs due to loss of deoxycytidine (dC) and ammonia [M−(dC+NH_3_)]^+^. Loss of dC and ammonia combined with loss of HN(CH_3_)CH = CH_2_ results in the ion at m/z 236.9 [M−(dC+NH_3_+HN(CH_3_)CH = CH_2_)]^+^. Loss of dC, ammonia and deoxyribose gives the ion at m/z 177.1 [M−(dC+NH_3_+deoxyribose)]^+^. The ion at m/z 177.1 can also be formed through homolytic cleavage of the glycosidic bond in the ion at m/z 294.3.

**Figure 6:**
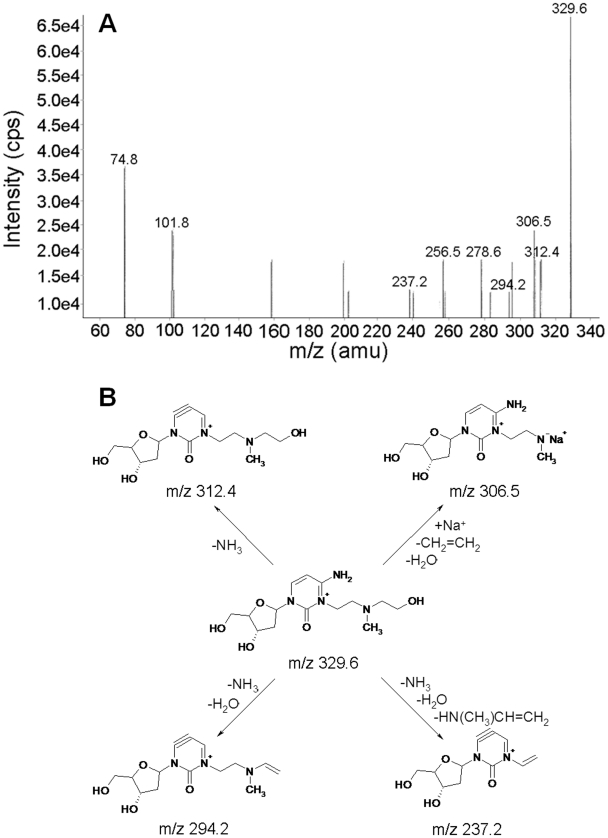
ESI-MS/MS analysis of N^3^dC-mech-OH. **A:** ESI-MS/MS spectrum of N^3^dC-mech-OH. **B:** Fragmentation pattern of N^3^dC-mech-OH. The IUPAC name for N^3^dC-mech-OH is [4-amino-3-[2-(2-hydroxyethyl(methyl)amino)ethyl]-1-[4-hydroxy-5-(hydroxymethyl)tetrahydrofuran-2-yl]pyrimidin-3-ium-2-one].

Based on possible hydrolysis of the N^3^dC-mech-N^3^dC crosslink, we also considered the presence of N^3^dC-mech-OH (Figure 6) in the ESI-MS/MS analysis. This species has an expected m/z of 329.2, and a signal at m/z 329.6 was detected. Fragmentation of [M]^+^ m/z 329.6 gave product ions with m/z 312.4 [M−NH_3_]^+^, m/z 294.2 [M−(NH_3_+H_2_O)]^+^, m/z 306.5 [M−(H_2_O+CH_2_ = CH_2_)+Na)]^+^, and m/z 237.2 [M−(NH_3_+H_2_O+HN(CH_3_)CH = CH_2_)]^+^. The product ions with m/z 237.2 and 294.2 are identical to the respective ions with m/z 236.9 and 294.3 obtained in fragmentation of the N^3^dC-mech-N^3^dC molecular ion. Therefore, the presence of N^3^dC-mech-OH and its fragmentation pattern supports the conclusion that the dC-mech-dC crosslink forms through cytosine N^3^.

## Discussion

The key results in the ESI-MS/MS analysis of the mechlorethamine-crosslinked DNA were the appearance of a doubly charged molecular ion and fragmentation of this ion with neutral loss of ammonia, consistent with the presence of an unreacted exocyclic amino group at N^4^. This fragmentation is in contrast to that observed for benzo[a]pyrene-7,8-dihydrodiol-9,10-epoxide DNA adducts formed at amino groups on guanine, cytosine and adenine, which gave no product ions with loss of ammonia [35]. Our observed loss of ammonia is also inconsistent with the imine that would be formed at N^4^ following crosslinking through O^2^. The absence of a molecular ion at m/z 538 further indicates that the mechlorethamine C-C crosslink does not form through N^4^ or O^2^. In addition, the heterolytic cleavage to give [dC−CH = CH_2_]^+^ m/z 236.9 is consistent with similar cleavage of mechlorethamine crosslinks from guanine to adenine [34]. Collectively, these results show that mechlorethamine crosslinks a C-C mismatch pair in a DNA duplex through cytosine N^3^.

The absence or presence of loss of ammonia (17 mass units) in fragmentation of the ESI-MS/MS product ion has been used in several previous studies as evidence for base modification at a ring N atom (N^3^ in cytosine) or an exocyclic amino group (N^4^ in cytosine). Loss of ammonia is taken to indicate that the original base had an intact amino group, and thus the adduct formed at the ring N. On the other hand, the absence of loss of ammonia suggests that the amino group was modified in the adduct. Singh et al. showed that ethylation of guanine occurred at the ring N^7^ atom and not at exocyclic N^2^ based on observation of the loss of ammonia from ethylguanine [41]. Similarly, Chao et al. showed that methylation and ethylation of guanine occurred at the ring N^7^ atom [42]. In contrast, Gaskell et al. showed that the reaction of benzo[a]pyrene-7,8-dihydrodiol-9,10-epoxide (B[a]PDE) with 2′-deoxynucleoside-3′-monophosphates occurred at the exocyclic amino group of each base, based on the observation of no loss of ammonia in MS fragmentation of the reaction products [35]. These findings should also be viewed in the context of the results obtained by Cao and Wang for fragmentation of the protonated ions of 2′-deoxycytidine and 5-substituted 2′-deoxycytidine selectively labeled with ^15^N atoms [43]. Substitution at position 5 influenced the fragmentation pattern and ammonia was lost from either the exocyclic N^4^ or ring N^3^ due to exchange of nitrogen between these positions [43]. These results suggest that care is required with identification of modifications on N^4^ or N^3^ of cytosine by MS/MS. This is an important caveat in the current work, but we note that the nitrogen exchange occurred with cytosine modified at position 5 and without modifications on the exocyclic N^4^ or ring N^3^ atoms [43]. Loss of ammonia from a cytosine base with modification at N^3^ gave fragmented ions containing a heteroaryne skeleton. The presence of six-membered rings with a triple bond such as heteroarynes and arynes (e.g. benzyne) is commonly found in ESI-MS/MS fragmentation [49]–[52].

It is possible that the crosslink formed through N^4^ or O^2^ could undergo spontaneous double protonation in the mass spectrometer to form a doubly charged molecular ion. However, it is likely that the formation of a molecular ion with a single charge would occur more easily than that with a double charge, and the MS spectra should then show both [M+H]^+^ and [M+2H]^2+^ signals at m/z 538.2 and 269.6, respectively. However, the presence of an ion close to m/z 269.6 and the absence of an ion close to m/z 538.2 make crosslink formation through N^4^ or O^2^ unlikely. On this basis, we also exclude a possible asymmetric mechlorethamine crosslink formed through N^3^ and O^2^, N^3^ and N^4^ or N^4^ and O^2^. In addition, the positive ion ESI-MS/MS product ion scan mode did not detect any m/z units that corresponded to other possible mechlorethamine-crosslinked dinucleosides, such as dG-mech-dG ([M+H]^+^, m/z 619.28; [M]^2+^, m/z 309.6) and dA-mech-dA ([M+H]^+^, m/z 587.29; [M]^2+^, m/z 293.64). This is due to the lack of suitable sites for these crosslinking reactions in our designed duplex. For example, the duplex lacks a 1,3 G-G interstrand crosslinking site, which is a favorable site for the mechlorethamine crosslinking reaction [22], [23]. The signal at m/z 329.6 was assigned to N^3^dC-mech-OH, which may have formed by aqueous hydrolysis of the N^3^C-mech-N^3^dC crosslink either during or after enzymatic digestion of the crosslinked duplex.

In the HPLC purification of the crosslink, use of a column temperature of 33°C and a slow increase in the amount of acetonitrile over 60 min was able to resolve the dC-mech-dC crosslink from single-stranded DNA. These chromatographic conditions were optimized in an earlier study [53]. The conditions are also sufficient to denature the DNA duplex because the presence of the C-C mismatch pair results in a low melting temperature (Tm). This is advantageous in the HPLC separation, since the duplex peak could have a similar retention time to that of the crosslink peak. The percentage of mechlorethamine-crosslinked C-C mismatch DNA detected by this method (about 25%) was in good agreement with our previous results for this reaction based on detection by gel electrophoresis [20], [21].

Enzymatic hydrolysis of the mechlorethamine-crosslinked DNA by SVPD and CIP was monitored using HPLC coupled with UV detection. The use of HPLC-UV for monitoring the progress of enzymatic digestion is useful for optimization of the reaction conditions (e.g. reaction time and temperature), including the suitable amount of SVPD and CIP. The crosslinked DNA was degraded to single nucleosides within 48 h, based on the similarity of the chromatogram with those for control sequences and the free DNA duplex, suggesting the complete hydrolysis of crosslinked DNA. The appearance of additional peaks on the chromatogram of the digested mixture indicated the presence of reaction products of mechlorethamine and DNA duplex. The HPLC chromatogram for the enzymatic digestion mixture of the crosslinked duplex showed two new peaks at 10.72 and 11.45 min. However, we detected only one crosslink species that gave an ion of m/z 269.2, corresponding to N^3^dC-mech-N^3^dC. Therefore, we are currently unable to define which of the peaks at 10.72 or 11.45 min corresponds to the ion of m/z 269.2.

Applications of mass spectrometry to analysis of DNA adducts are becoming more common. Enzymatic digestion coupled with HPLC-ESI-MS/MS analysis provides a basis for quantitative detection and characterization of small amounts of DNA adducts in vitro and in vivo [38]. Synthesis of an authentic standard for comparison of the fragmentation pattern is also useful. We have attempted synthesis of the N^3^dC-mech-N^3^dC crosslink for this purpose and for spectroscopic analysis, but formation of the species has proven to be difficult. This may be due to the requirement for multiple reactions (i.e. the need for reactions of two cytosine bases with one mechlorethamine) and the rapid hydrolysis of mechlorethamine. In addition, small-molecule synthetic methods may not appropriately mimic oligonucleotide secondary structure. Some nucleophilic groups in oligonucleotide bases may be hydrogen bonded, making them less available for reaction in comparison with individual bases with less hydrogen bonding. This may be a common problem for other adducts, and the growing number of DNA crosslinks for which the fragmentation pattern has been established should improve the reliability of future analyses of unknown adducts. Therefore, the fragmentation pattern of the mechlorethamine C-C crosslink reported here provides an important addition to the literature, as the first example of ESI-MS/MS analysis of a DNA adduct at the N^3^ position of cytosine.

## Materials and Methods

### Chemicals

Synthetic 15-mer oligodeoxynucleotides were purchased from Sigma-Proligo (St. Louis, MO). Mechlorethamine hydrochloride and monodeoxynucleosides (dA, dT, dG and dC) were also purchased from Sigma. Snake venom phosphodiesterase (SVPD, from *Crotalus adamanteus*) and calf intestinal phosphatase (CIP) were purchased from Sigma and Finnzyme (Espoo, Finland), respectively. Other reagents were of AR or HPLC grade.

### Preparation of the mechlorethamine-DNA crosslink

A DNA duplex solution (10 µM) in 0.1 M sodium chloride and 0.05 M Tris buffer pH 7.5 was prepared using stock solutions of d[CTCACACCGTGGTTC] (top strand, 100 µM, MW = 4505) and d[GAACCACCGTGTGAG] (bottom strand, 100 µM, MW = 4603). The solution was heated at 90°C for 2 min and then slowly cooled to room temperature to allow annealing of the DNA to form a duplex (MW = 9108) containing a C-C mismatch pair (the underlined bases in the two strands form this pair). The DNA duplex was also designed to avoid the presence of a site for formation of a mechlorethamine 1,3 G-G crosslink [22], [23]. The duplex solution (100 µl) was then incubated with 0.1 M mechlorethamine in dimethylsulfoxide (1 µl) for 2 h at room temperature to allow the reaction to go to completion [19]–[21].

### Purification and characterization of the mechlorethamine-DNA crosslinked duplex

The crosslinked duplex was purified by HPLC (Shimadzu-VP, Kyoto, Japan) using a Biobasic-C4 column (4.6×250 mm, 5 µ) (Thermo Electron, Waltham, MA) with a column temperature of 33°C. Optimization of the conditions of HPLC purification for the crosslink is described elsewhere [49]. Gradient elution was performed with 5–15% acetonitrile (ACN) in 100 mM TEAA and 0.1 mM EDTA over 60 min at a flow rate of 1 ml/min with UV detection at 260 nm. The injection volume was 20 µl. Control experiments were performed under the same HPLC conditions by injecting top-strand DNA, bottom-strand DNA, or annealed DNA duplex.

The percentage of DNA crosslinked by mechlorethamine was calculated from the peak area of the crosslink divided by the sum of all peaks in the chromatogram multiplied by 100. The HPLC-purified crosslinked duplex was obtained by collecting the appropriate eluting peak and desalting by ultracentrifugal filtration (Microcon® YM-3; Millipore, Billerica, MA) at 5000 rpm for 45 minutes. The purified crosslink solution was then freeze-dried and subjected to molecular weight determination by Autoflex II MALDI-TOF-MS (Bruker Daltonics, Billerica, MA) using the linear positive mode. Data processing was performed with Flex Analysis software. The sample was prepared by dispersing the purified crosslink in a matrix consisting of 3-hydroxypicolinic acid in acetonitrile/H_2_O (1/1, v/v) and ammonium acetate solution (12 mg/ml) at the volume ratio of 1∶1.

### Enzymatic digestion of the mechlorethamine-crosslinked DNA duplex

The purified crosslinked duplex was dissolved in water at 10 µM and 6.6 µl of this solution was combined with magnesium chloride (10 mM, 0.67 µl), Tris buffer pH 8.1 (50 mM, 0.67 µl), SVPD (0.4 units), and CIP (20 units). The resulting solution was adjusted to 100 µl with distilled water and incubated at 37°C for 48 h. After incubation, the enzymes were removed by ultracentrifugal filtration (Microcon® YM-3) at 5000 rpm for 45 minutes. Control experiments were performed using the same conditions with 6.6 µl of 10 µM top-strand DNA, 10 µM bottom-strand DNA, 10 µM DNA duplex, or 100 µM polydeoxynucleotides (15mer sequences of poly-dA, poly-dT, poly-dG and poly-dC).

Formation of the products of digestion of the mechlorethamine-crosslinked duplex and the control DNAs was monitored by injecting 20 µl the digested products after ultracentrifugal filtration into HPLC (Shimadzu-VP) using a Rainin Microsorb-C18 column (4.6×150 mm, 5 µ) (Varian, Lake Forest, CA) with the column temperature at 25°C. Gradient elution was performed with 2–20% ACN in 50 mM sodium phosphate buffer (pH 5.8) over 20 min at a flow rate of 1 ml/min with UV detection at 260 nm.

### ESI-MS/MS analysis of the mechlorethamine C-C crosslink

The crosslink species produced by enzymatic digestion of the mechlorethamine-crosslinked duplex was analyzed using an API 4000 triple quadrupole instrument (Applied Biosystems, Foster City, CA) with a turbo-ion spray source using electrospray ionization in positive ion mode. The ion source conditions were an ion spray voltage of 4500 V. Nitrogen was used as nebulizing gas (20 psi) and curtain gas (10 psi). The filtrate after ultracentrifugal filtration of the enzymatic digest of the mechlorethamine-crosslinked duplex was diluted with purified water and directly infused into the mass spectrometer at a flow rate of 10 µl/min. The scan range was m/z 50–600. The selected ion was isolated and fragmented by collisions with nitrogen (4 psi). Data processing was performed using Analyst software (version 4.4.2). Signals with appropriate values of m/z corresponding to a mechlorethamine C-C crosslink or hydrolytic products of the crosslink were selected and fragmented. The resulting MS spectra were analyzed to determine the connectivity. IUPAC names for the fragments were obtained using Symyx® Draw Vervion 3.2 (Symyx Technologies, CA, USA).
